# AI-driven low-cost rehabilitation exergame as a lightweight framework for stroke assessment

**DOI:** 10.1038/s41746-026-02383-1

**Published:** 2026-01-28

**Authors:** Júlia Tannús, Caroline Valentini, Eduardo Naves

**Affiliations:** Assistive Technologies Group, Federal University of Uberlândia, Uberlândia, Brazil

**Keywords:** Computational biology and bioinformatics, Engineering, Health care, Mathematics and computing, Medical research, Neurology, Neuroscience

## Abstract

Stroke is a leading cause of long-term disability, often affecting upper-limb motor function and requiring continuous assessment. The Fugl-Meyer Assessment (FMA), though a clinical gold standard, is time-consuming and demands specialized personnel. This study presents an AI-driven, low-cost rehabilitation exergame that simultaneously provides therapy and automatically estimates upper-limb motor performance during gameplay using only a standard camera. Sixteen kinematic and spatiotemporal features were extracted from 2D hand and arm trajectories of twelve post-stroke individuals (24 limbs, 14 affected) using the MediaPipe framework. Features such as hand angle, range of motion, movement area, traveled distance, and shoulder–elbow coordination showed strong correlations with FMA scores and stratified participants by motor severity. A lightweight linear regression model achieved high predictive performance (Spearman *ρ* = 0.92, R² = 0.89, RMSE = 4.42) and classified severity levels with 86–93% accuracy. This interpretable approach outperformed complex machine learning models, highlighting the clinical relevance of transparent metrics embedded in gameplay. The proposed framework is sensor-free, scalable, and reproducible, offering immediate feedback while reducing clinical workload and enabling accessible digital biomarkers for telerehabilitation and remote monitoring after stroke.

## Introduction

Stroke remains a leading cause of long-term disability worldwide, with over 12 million new cases annually and approximately 100 million stroke survivors globally, many of whom experience upper-limb motor deficits that hinder daily function^1,2^.

For rehabilitation, while in-person treatments remain essential, they can be time-consuming, resource-intensive, and limited in availability^3^. These programs can also be tedious and financially burdensome, requiring patient transportation to clinical sites. In contrast, video game-based rehabilitation has emerged as a motivational and accessible alternative^4,5^. Virtual reality games have proven capable of engaging stroke patients in repetitive motor tasks that support neuroplasticity and motor recovery^6–9^.

In parallel, accurate assessment of motor impairment is essential for treatment planning and recovery monitoring. The Fugl-Meyer Assessment (FMA) is widely considered a gold standard for quantifying post-stroke motor function^10–12^, but it is time-consuming, subjective, and requires trained clinicians^3,13,14^.

For these reasons, there are some initiatives to automate motor assessments, including smartphone apps^15,16^, motion sensor-based systems^17,18^, and machine learning models that estimate motor scores from movement features^19–21^.

While many of these systems are promising, they often require dedicated assessment time, lack interpretability, need external sensors (such as depth cameras or inertial sensors), or rely on complex architectures such as 3D pose estimation or deep neural networks^5,22^. In contrast, combining motor assessment directly into the rehabilitation game itself, as in the present work, offers a compelling advantage: it eliminates the need for separate clinical evaluations, reduces monotony, increases engagement, and enables real-time, high-frequency tracking of recovery without burdening therapists or patients. This integration promotes scalability and personalization, allowing stroke survivors to be continuously monitored during gameplay using simple, transparent metrics that reflect functional performance.

Unlike conventional assessments that must be administered separately, the proposed system performs evaluation during therapeutic gameplay itself. As the patient engages in motor exercises, the same movement data used for rehabilitation are automatically analyzed to generate FMA-equivalent digital scores, eliminating the need for additional assessment time.

In this framework, the term “AI-driven” refers to the use of Google’s MediaPipe: a computer-vision framework powered by machine learning for real-time hand and body tracking. By leveraging this AI-based system, the same standard camera used for gameplay also performs motion capture, removing the dependence on specialized hardware. The resulting kinematic features serve as AI-driven digital biomarkers, offering objective and transparent indicators of motor function while maintaining a low-cost and scalable setup.

Therefore, this work proposes a lightweight, AI-based exergame system for post-stroke rehabilitation that also estimates upper-limb motor function using only simple, interpretable kinematic features extracted from 2D wrist and hand movements during gameplay. In this paper, it was tested how well these features reflect clinical severity, discriminate across FMA strata, and predict motor scores. By leveraging basic movement metrics in a linear equation, such as hand aperture, spatial exploration, and joint coordination, without relying on expensive hardware or black-box deep learning models, this system offers a cost-effective and accessible alternative to traditional assessments.

## Results

### Participant characteristics

Twelve individuals with chronic stroke participated in the study, including nine males and three females. Participants ranged in age from 21 to 66 years, with a mean age of 46.7 years (SD = 13.2). The time since stroke ranged from four months to 30 years. Most participants had experienced ischemic stroke (*n* = 10), while two had suffered hemorrhagic stroke. In two cases, bilateral upper-limb paresis was reported.

Each participant performed gameplay sessions using both upper limbs and was assessed using the FMA on each side. This resulted in a total of 24 limb-level observations, enabling intra-individual comparisons. Table 1 presents the demographic and clinical characteristics of the participants, including age, gender, affected side, and FMA scores for each limb.

**Table 1:** Details of the stroke subjects

Subject	Age (years)	Gender	Affected Side	FMA^a^ Left	FMA^a^ Right
S1	66	Male	Left	23	62
S2	36	Male	Left	15	66
S3	33	Female	Right	66	62
S4	53	Male	Both	48	29
S5	45	Male	Both	26	17
S6	21	Male	Right	66	33
S7	46	Male	Left	43	66
S8	49	Female	Left	22	66
S9	61	Female	Left	21	66
S10	37	Male	Left	48	66
S11	52	Male	Left	13	66
S12	61	Male	Right	66	15

^a^FMA refers to the Fugl-Meyer Assessment Upper Extremity, a score between 0 (no function) and 66 (intact).

Based on FMA scores, the tested limbs were stratified into four categories: (i) severe impairment (FMA ≤ 20), (ii) moderate impairment (21 ≤ FMA ≤ 45), (iii) mild impairment (FMA > 45), and (iv) control limbs (non-affected side of participants with unilateral hemiparesis). Table 2 summarizes the distribution of upper limbs across these severity levels, including the number of limbs per group, average age, and mean FMA scores.

**Table 2:** Upper limbs tested stratified by severity level

Level of Hemiparesis	Number of Limbs	Left Side	Average Age (years)	Average FMA Score
Severe	4	2	48.5 ± 10.6	15.0 ± 1.6
Moderate	7	5	50.3 ± 7.4	28.0 ± 10.2
Mild	3	2	41.0 ± 10.6	52.7 ± 8.1
Control	10	3	39.0 ± 19.1	65.0 ± 2.0

Each participant contributed two upper limbs to the dataset (one left and one right), for a total of 24 limbs. Limbs were classified according to their motor function based on the FMA: (i) Severe: FMA ≤ 20, (ii) Moderate: FMA 21-45, (iii) Mild: FMA > 45 (affected limb), and (iv) Control: non-affected limb.

### Feature summary

**Fig. 1:**
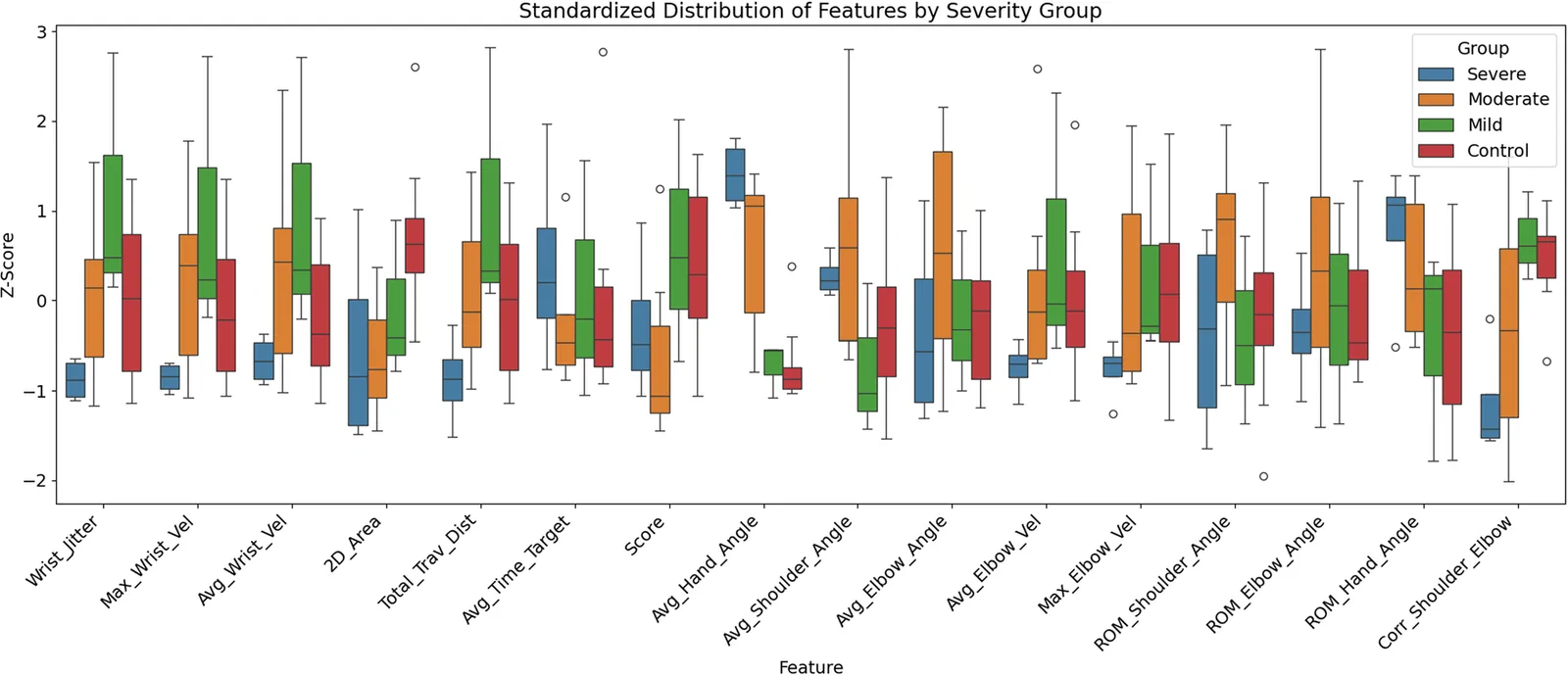
Standardized distributions of gameplay-derived features stratified by upper-limb severity level. The plot shows Z-scores for all 16 kinematic features and the in-game score, grouped by severity. Features such as Avg_Hand_Angle, ROM_Hand_Angle, Corr_Shoulder_Elbow, and 2D_Area demonstrate clear separation between groups, with progressively higher median values from Severe to Control. Boxes represent interquartile ranges (IQR), with whiskers indicating 1.5×IQR and dots representing outliers. For each feature, the degree of separation between the boxes indicates how well that variable discriminates among severity categories: the less overlap, the better its classification capability. The direction of the median values (whether they increase or decrease across groups) shows whether the relationship with motor function is linear and positive or negative, that is, whether higher values represent better or worse performance.

Gameplay-derived features were standardized (Z-scores) and compared across four severity groups (Severe, Moderate, Mild, Control). Figure 1 presents a boxplot summarizing the distribution of all 16 features across these categories.

Several features demonstrated consistent and discriminative trends. Specifically, *Avg_Hand_Angle*, *ROM_Hand_Angle*, *Corr_Shoulder_Elbow*, and *2D_Area* exhibited progressive and monotonic changes in median values across severity levels, with *Avg_Hand_Angle* standing out as the most clearly stratified variable.

Conversely, features such as *Avg_Wrist_Vel*, *ROM_Shoulder_Angle*, and *Avg_Elbow_Angle* showed minimal group separation and high within-group dispersion, indicating limited discriminative power in isolation.

Overall, the feature distribution analysis reveals three key conclusions:

1. Certain features consistently reflect clinical severity, particularly *Avg_Hand_Angle*, *ROM_Hand_Angle*, *Corr_Shoulder_Elbow*, and *2D_Area*, making them strong candidates for predictive modeling and digital assessment tools.
2. Some features, despite high variability, still provide partial stratification, suggesting they may be useful when combined into multivariate models.
3. Some metrics are uninformative and should be used cautiously.

**Fig. 2:**
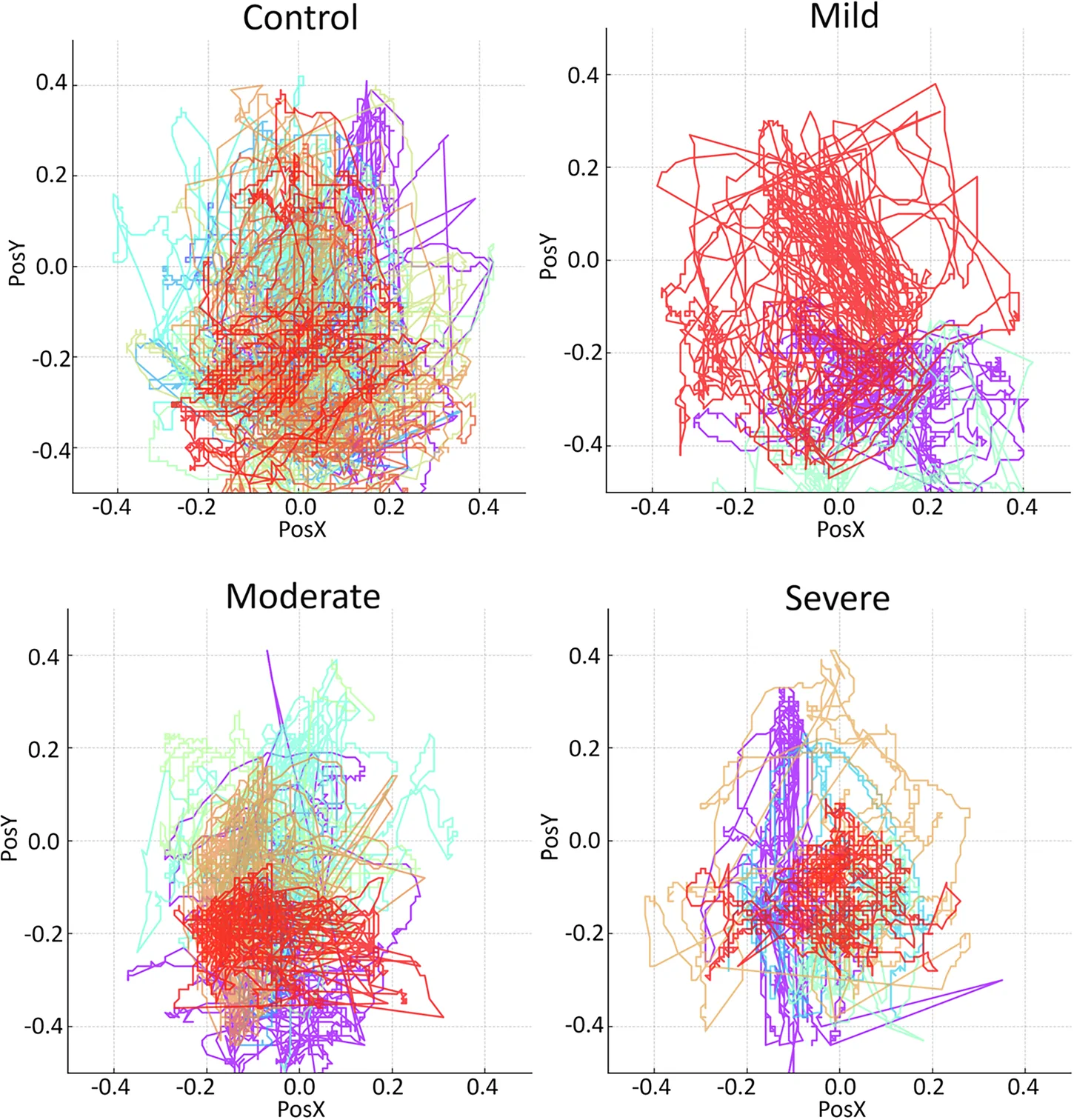
Frame-by-frame wrist trajectories captured during gameplay, stratified by motor severity level based on Fugl-Meyer scores. Limbs in the Control group explored a significantly larger portion of the 2D workspace, demonstrating well-distributed trajectories and full utilization of the screen area, supporting the discriminative power of the *2D_Area* feature.

Trajectory plots (Fig. 2) showed compact, centralized movements in the Severe group versus broader exploration in Controls, with Mild and Moderate groups presenting intermediate patterns. The results reinforce *2D_Area* as a clinically interpretable indicator of upper-limb functional reach in post-stroke individuals.

### Correlation with clinical score per feature

**Fig. 3:**
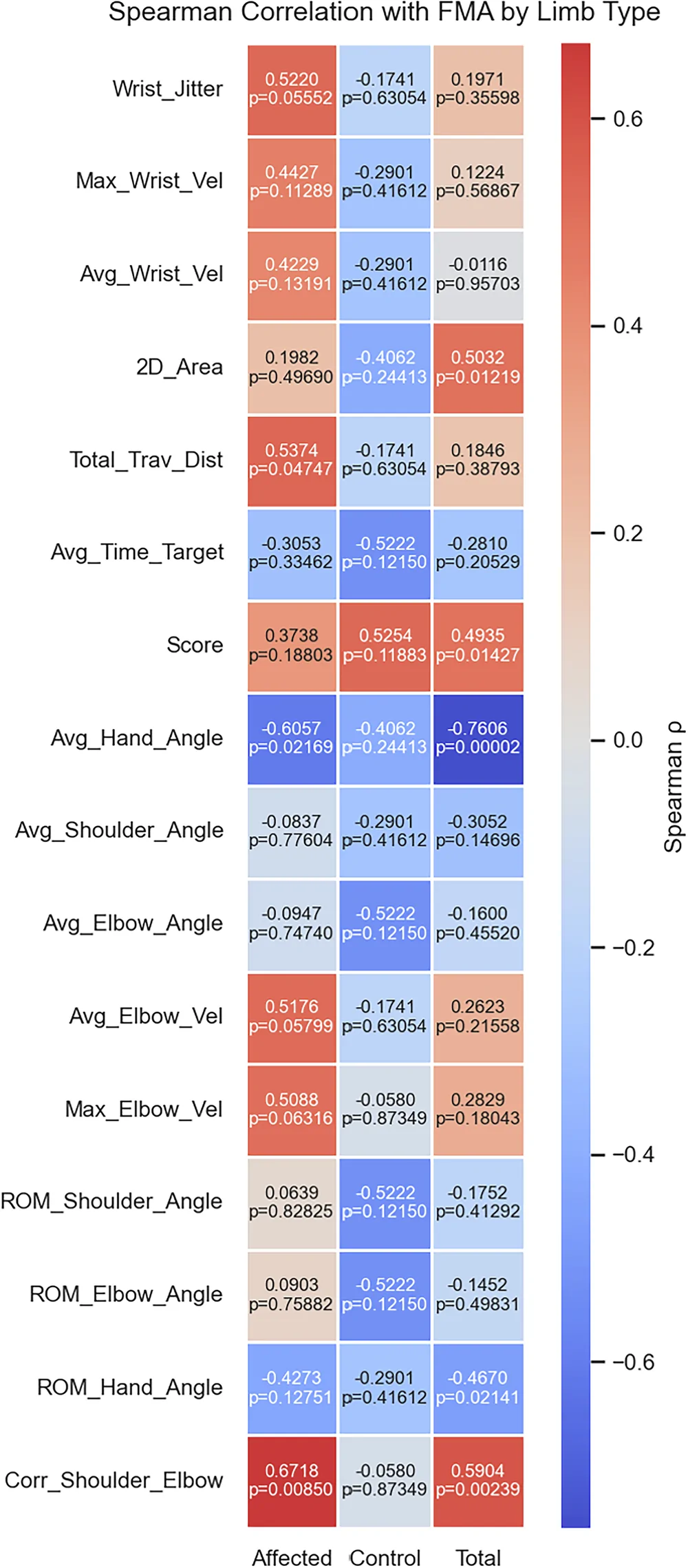
Spearman correlation coefficients (ρ) and corresponding *p*-values between kinematic features and FMA scores, stratified by limb type (Affected, Control, and Total). Cells are colored according to the magnitude and direction of ρ, with warmer tones indicating positive correlations and cooler tones indicating negative correlations. For example, a dark blue in the Avg_Hand_Angle group - total and a very warm red in the Corr_Shoulder_Elbow group - affected indicate the characteristics with the highest correlation.

To explore how gameplay-derived features relate to motor function, Spearman correlations were computed between each feature and the FMA score, stratified by limb type (affected, control), and considering all limbs together (total). The results are presented in Fig. 3.

The following features showed statistically significant correlation with FMA, ordered by the absolute value of Spearman’s ρ. Significant correlations are highlighted using: *(*p* < 0.05), ** (*p* < 0.01), and *** (*p* < 0.001):

1. Avg_Hand_Angle (total): *ρ* = −0.76 ***
2. Corr_Shoulder_Elbow (affected): *ρ* = 0.67 **
3. Avg_Hand_Angle (affected): *ρ* = −0.61 *
4. Corr_Shoulder_Elbow (total): *ρ* = 0.59 **
5. Total_Trav_Dist (affected): *ρ* = 0.54 *
6. 2D_Area (total): *ρ* = 0.50 *
7. Score (total): *ρ* = 0.49 *
8. ROM_Hand_Angle (total): *ρ* = −0.47 *

These results confirm that features related to joint coordination, hand orientation, trajectory coverage, and game performance are consistently associated with clinical motor scores. In particular, the Average Hand Angle and Shoulder-Elbow Coordination show robust associations across both affected limbs and the entire dataset.

### Predictive modeling

To explore the predictive capacity of the extracted features in estimating clinical motor function, multiple linear regression analyses were performed using exhaustive feature selection with all 16 features, with maximum of 5 features per equation, to avoid overfitting. This approach systematically evaluates all possible combinations of features to identify the subset that maximizes correlation with the ground truth FMA scores.

Separate models were constructed for the affected limb and for the total group. For each group, the best regression models were identified according to Spearman’s ρ, RMSE, and R². All models were derived from standardized (Z-scored) features. The top-performing equations, along with their corresponding performance values, are summarized in Table 3.

**Table 3:**
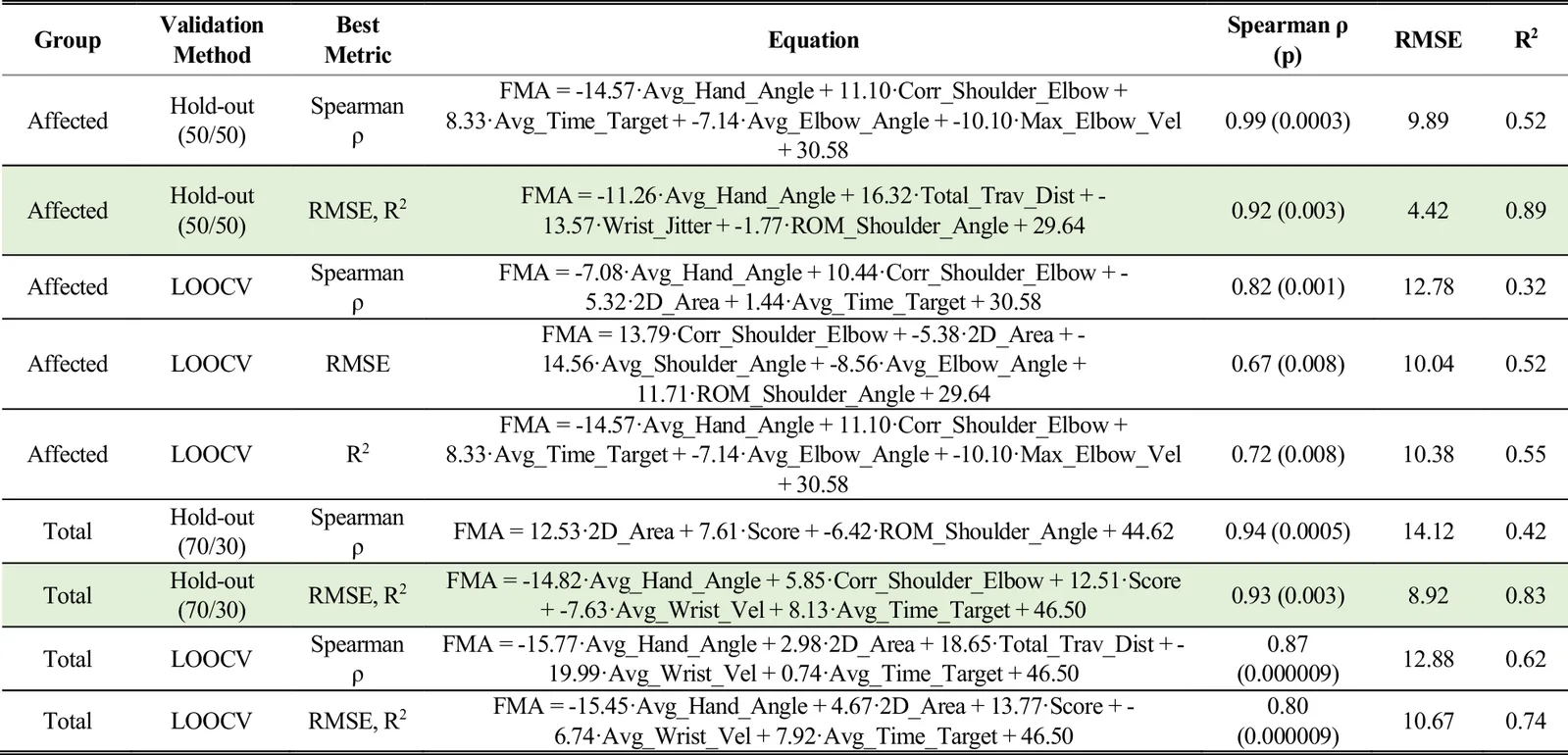
Best multiple linear regression models by limb group, validation method and best metric (Z-Score Normalized)

LOOCV Leave-one-out cross validation. The best overall models for the Affected (*n* = 14) and Total (*n* = 24) group are highlighted in green.

For the affected limb group, the optimal multiple regression models varied depending on the evaluation metric. Using a 50/50 hold-out split, the model maximizing Spearman correlation achieved an excellent association with the clinical score (*ρ* = 0.99, *p* = 0.0003), although with moderate explained variance (R² = 0.52) and RMSE = 9.89. By contrast, the model optimized for error reduction reached the best overall fit (R² = 0.89, RMSE = 4.42), still maintaining a strong correlation (*ρ* = 0.92, *p* = 0.003). LOOCV (leave-one-out cross validation) models for the affected subset produced lower correlations (*ρ* = 0.67–0.82) and explained variance (R² = 0.32–0.55), confirming that the hold-out strategy yielded more stable and clinically relevant predictions in this small sample.

For the total group, results were more consistent. The hold-out models performed robustly, with the best solution balancing high correlation (*ρ* = 0.94, *p* < 0.001) and strong explained variance (R² = 0.83) while maintaining an acceptable error (RMSE = 8.92). LOOCV models again performed slightly worse, particularly in terms of error, reinforcing the advantage of hold-out validation for this dataset.

Importantly, the features selected through exhaustive feature selection did not always correspond to those with the strongest individual correlations with the FMA. This highlights that exhaustive feature selection identified not only individually strong predictors but also synergistic feature sets that together maximized predictive performance. Accordingly, variations in coefficients across equations arise from the inclusion of different predictor combinations identified through exhaustive feature selection. These alternative formulations highlight multiple relevant feature sets rather than instability, reflecting the exploratory nature of this proof-of-concept study. Thus, while univariate correlations provided useful guidance, the multivariate regression models offered a more comprehensive view of how gameplay-derived metrics map onto clinical motor outcomes.

**Fig. 4:**
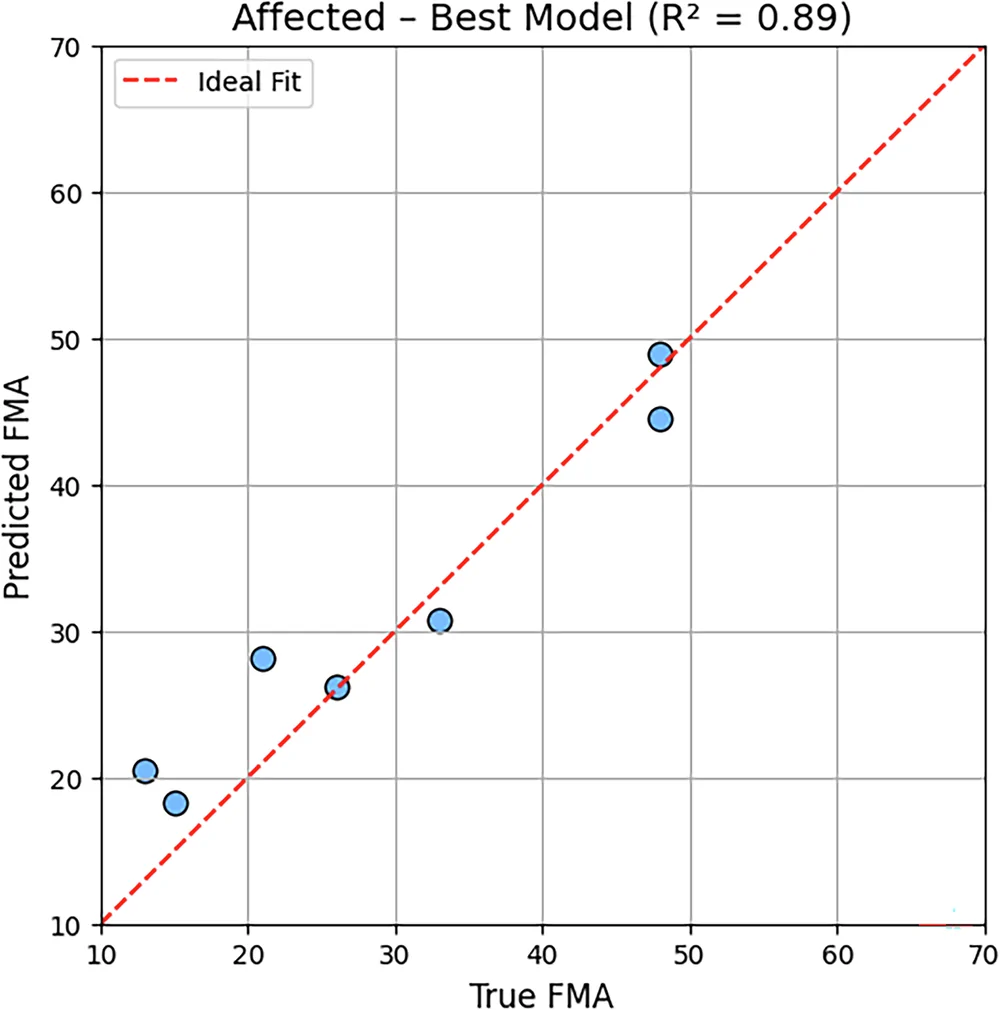
Predicted FMA values from the best multiple linear regression model for the affected limb subset. The red dashed line represents the ideal fit (y = x).

The final regression model for the affected group, summarized in Table 3 and illustrated in Fig. 4, achieved R² = 0.89 and Spearman’s *ρ* = 0.92, representing the best overall balance among the three performance metrics (R², RMSE, and Spearman correlation). Earlier exploratory models with higher ρ but lower R² were excluded from the final results to ensure consistency between rank and variance-based measures.

### Exploratory diagnostic accuracy

The confusion matrix in Table 4 demonstrates strong classification performance across severity levels. Moderate cases were most consistently recognized, with all 7 instances correctly classified. Mild cases showed agreement in 2 out of 3 participants, while severe cases were correctly identified in 3 out of 4 participants. Importantly, misclassifications occurred only between adjacent severity levels (e.g., mild vs. moderate, moderate vs. severe), with no extreme errors across non-neighboring classes.

**Table 4:** Confusion matrix by class (affected)

	Predicted				
		Mild	Moderate	Severe	Total
True	Mild	2	1	0	3
	Moderate	0	7	0	7
	Severe	0	1	3	4
	Total	2	9	3	14

As summarized in Table 5, the regression equation achieved balanced performance across all groups. The Mild class obtained perfect precision (1.00) but slightly lower recall (0.67), resulting in an F1-score of 0.80 and overall accuracy of 0.93. The Moderate class showed the highest recall (1.00) with good precision (0.78), achieving an F1-score of 0.88 and accuracy of 0.86. The Severe class also reached high performance, with precision of 1.00, recall of 0.75, and an F1-score of 0.86, corresponding to an accuracy of 0.93. These results indicate that the regression-based model generalized well across severity categories, reliably distinguishing between mild, moderate, and severe impairment levels.

**Table 5:** Best regression equation performance by class (affected)

Class	Precision	Recall	F1-Score	Accuracy
Mild	1.00	0.67	0.80	0.93
Moderate	0.78	1.00	0.88	0.86
Severe	1.00	0.75	0.86	0.93

### Machine learning analysis

The Random Forest model demonstrated robust predictive capacity when trained on the total dataset, achieving *ρ* = 0.76 (*p* = 0.030), R² = 0.63, and RMSE = 12.25 FMA points in hold-out validation.

The temporal deep learning model (CNN1D+BiLSTM) achieved modest performance on the independent arm-level test set (R² = 0.59, RMSE = 11.5, *ρ* = 0.75; *p* = 0.05), underscoring the challenge of generalization with small datasets despite promising validation results.

Regularized linear models yielded highly interpretable sparse solutions. Both Lasso (*α* = 7.05) and Elastic Net converged to the same two predictors: *Score* (positive coefficient) and *Avg_Hand_Angle* (negative coefficient), and achieved comparable performance (R² = 0.53, RMSE = 13.7, *ρ* = 0.83; *p* = 0.011). Although they explained slightly less variance than Random Forest, their strong monotonic association with FMA and parsimony make them particularly appealing for clinical translation. Summarized results are in Table 6. Importantly, the best result did not surpass the best linear regression models.

**Table 6:** Summary of machine learning models applied (total group)

Model	R²	RMSE	Spearman ρ
Random Forest	0.63	12.25	0.76 (*p* = 0.030)
CNN1D+BiLSTM	0.59	11.5	0.75 (*p* = 0.05)
Lasso (α ≈ 7.05)	0.54	13.7	0.83 (*p* = 0.011)
Elastic Net	0.53	13.8	0.83 (*p* = 0.011)

This evaluation was done in the total group, because the affected group did not wield statistically significant results, due to low sample size, which also highlights the regression model superiority.

Machine learning models were included as exploratory analyses to provide contextual comparison, and not as the primary or final methodological objective of the study. They were not prioritized due to their limited feasibility for real-time deployment within a mobile exergame environment.

## Discussion

This study evaluated the feasibility and clinical relevance of an AI-driven, low-cost rehabilitation exergame for assessing upper-limb motor function in individuals with chronic stroke. The results demonstrated that gameplay-derived kinematic features not only reflect clinically interpretable differences across severity levels but also predict the gold-standard FMA scores with high accuracy, highlighting the system’s potential as a transparent and scalable digital biomarker.

The exergame simultaneously serves therapeutic and evaluative purposes, capturing clinically interpretable kinematic data during gameplay without requiring separate assessment sessions. This dual function enhances efficiency and allows objective, high-frequency monitoring of recovery progress.

Identifying features that correlate with FMA is essential for building interpretable and clinically interpretable assessment tools. In this study, several metrics stood out for their discriminative power across severity levels.

Average hand angle and hand angle range of motion were particularly informative, capturing the ability to open the hand, a key clinical deficit in severe cases, often associated with clenched hands or spasticity. Their progressive changes across severity groups reinforce hand aperture as a central marker of motor impairment. In the regression equations, the negative coefficient of *Avg_Hand_Angle* indicates that increased finger flexion (clenched-hand posture) is associated with lower FMA scores, consistent with clinical patterns of spasticity. Conversely, positive coefficients such as those for *Corr_Shoulder_Elbow* and *2D_Area* denote more coordinated and spatially extensive movements, which align with better voluntary control and higher functional scores. These relationships strengthen the interpretability of the model and its translational relevance for clinical assessment.

Spatial exploration features, such as 2D movement area and total traveled distance, also showed strong associations with FMA, reflecting how higher-functioning participants performed broader, more frequent movements. Shoulder-elbow coordination further distinguished between synergy-dominated patterns in low-FMA patients and refined joint control in those with better outcomes.

Interestingly, features traditionally used in motor assessment, such as velocity or raw joint angles, were not among the best correlated features. This may be due to clinical heterogeneity: patients may be spastic or flaccid, limiting the generalizability of angular metrics. Velocity, in particular, proved ambiguous, since high values may reflect either controlled speed in higher-functioning participants or poorly coordinated, abrupt movements in those with impairments. As a result, speed alone did not reliably indicate motor capacity. These findings indicate that features that capture core motor mechanisms can be more valuable than less interpretable metrics.

Multiple linear regression models built on the most relevant features achieved highly accurate predictions of FMA scores, particularly in the affected limb group (*ρ* = 0.92, RSME = 4.42, R² = 0.89). Even with hold-out validation, the models retained strong predictive power, confirming that motor function can be reliably inferred from simple combinations of movement metrics.

Notably, more complex machine learning approaches, including Random Forest and temporal deep learning models (CNN1D+BiLSTM), did not surpass the performance of the lightweight linear regression. These advanced models showed promising internal validation but generalized poorly on independent test sets, underscoring the challenge of applying black-box architectures to small datasets. Regularized linear models such as Lasso and Elastic Net offered interpretability but achieved lower accuracy than the proposed regression approach.

This finding highlights a central innovation of the framework: a simple, interpretable functional evaluation algorithm, directly integrated into post-stroke rehabilitation gameplay, can outperform more complex machine learning pipelines, making the system more reliable, clinically transparent, and easier to adopt in real-world. This design ensures low computational cost, allowing on-device analysis and continuous patient monitoring during rehabilitation sessions. The ability to assess motor function as the patient plays removes the need for standalone assessment software and supports more frequent, naturalistic evaluations.

The small cohort size limited the ability to distinguish performance differences among machine learning models. However, this constraint also highlights a practical advantage of the proposed lightweight regression model, which achieved strong correlations and clinically relevant predictions with relatively few training samples.

A larger, follow-up study is planned to expand the dataset and validate these exploratory findings, enabling robust benchmarking across models trained with homogeneous data representations.

To contextualize our findings, Table 7 summarizes representative studies that used different sensing modalities and modeling approaches for automated upper-limb motor assessment. Most previous systems rely on specialized hardware such as depth cameras, inertial sensors, or multi-sensor fusion, and often apply black-box architectures that hinder interpretability. In contrast, the present framework achieves comparable correlations with clinical scores using only the standard RGB camera from the display device (e.g., laptop, tablet) and a transparent regression model embedded directly within gameplay.

**Table 7:** Comparison of related studies (fma-ue 33 items)

Study	Sensor Type	Modeling Approach	Dataset Size	Main Result
Song et al.^26^	Camera and inertial sensors of a smartphone	Decision trees	*n* = 10	R² = 0.78
Chen et al.^20^	Custom optical capture device	Decision trees	*n* = 79	RMSE = 17.4
Jiang et al.^27^	Microsoft Kinect	Fuzzy inference	*n* = 25	Accuracy = 93.5%
Present study	Camera of the game display device (MediaPipe) – no external sensors	Linear regression	*n* = 24	R² = 0.89

This combination of AI-based motion capture and lightweight, interpretable evaluation demonstrates how accessible, low-cost solutions can provide clinically interpretable digital biomarkers for telerehabilitation.

The exploratory diagnostic accuracy analysis further demonstrated that predicted FMA values could stratify patients into clinical severity groups with 86–93% accuracy, misclassifying only between neighboring categories. This level of agreement is clinically acceptable and reinforces the framework’s potential to support automated functional classification.

Unlike previous systems requiring Kinect, Wii, depth cameras, inertial sensors, or exoskeletons, the proposed exergame achieves assessment using only a standard camera. By embedding evaluation into gameplay itself, the system eliminates the need for additional software or separate testing sessions. This sensor-free, low-cost, and scalable approach reduces setup complexity, enhances patient engagement, and lowers clinical workload, paving the way for widespread telerehabilitation applications.

This study introduced a lightweight, AI-driven rehabilitation exergame capable of simultaneously engaging patients in therapy and assessing upper-limb motor function. Specific gameplay-derived features, particularly hand aperture, 2D movement area, and shoulder-elbow coordination, showed strong associations with the FMA, enabling accurate prediction of clinical scores and stratification of motor severity.

Crucially, a simple linear regression model provided superior performance and interpretability compared to more complex machine learning approaches, demonstrating that transparency and clinical usability can outweigh the marginal gains of black-box algorithms in small-scale rehabilitation studies.

The framework’s low-cost and sensor-free design is a major innovation: by relying solely on a standard camera, it avoids the expense and logistical burden of external sensors, such as Kinect, inertial units, or exoskeletons. Furthermore, because assessment occurs in real time during gameplay, the system eliminates the need for dedicated evaluation sessions or post-processing software, offering immediate feedback and reducing clinician workload.

Taken together, these features position the proposed exergame as a practical, scalable, and innovative tool for stroke rehabilitation and remote monitoring. While the small sample size and cross-sectional design limit generalizability, this proof-of-concept study demonstrates strong potential for integration into telerehabilitation settings. Future work should validate the framework in larger and more diverse populations, explore longitudinal responsiveness to recovery, and investigate its role in personalized rehabilitation pathways. Also, multiple independent raters and inter-rater reliability analyses will be included to enhance robustness.

## Methods

### Exergame system

**Fig. 5:**
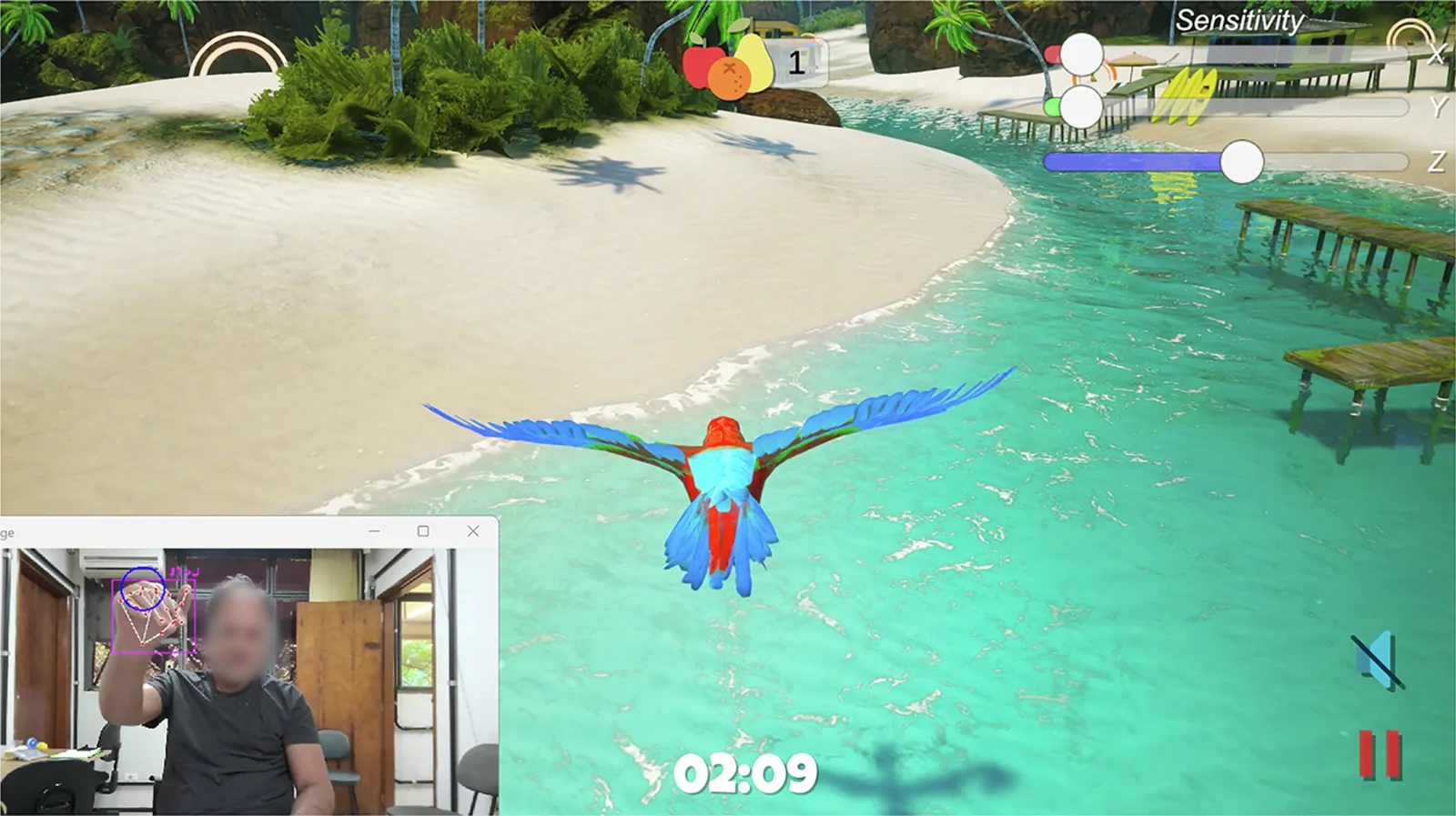
Screenshot of the exergame during gameplay. The main scene displays the bird avatar navigating a tropical environment while collecting virtual fruits. The participant’s wrist position in the X and Y axes (in pixels), captured in real-time via MediaPipe hand tracking, is used to control the character’s movement. Source: The authors. Written consent to publish the image was obtained from the participant.

The exergame employed in this study was developed using the Unity 2020.1.17 game engine (https://unity.com/) and offers a dynamic and engaging environment aimed at evaluating upper-limb motor function. The gameplay centers around guiding a bird avatar to collect fruits dispersed across a virtual landscape (Fig. 5). The game was created to entertain users and enable rehabilitation, not to resemble a clinical evaluation.

Player control is based on real-time 3D hand tracking, implemented through the MediaPipe framework^23^, which operates with a standard camera, commonly found on devices such as tablets, laptops, and smartphones.

MediaPipe relies on Artificial Intelligence-based models for real-time hand and body landmark detection, using deep neural networks trained on large-scale human pose datasets. This AI-driven architecture enables accurate 3D tracking directly from standard RGB images, allowing the same low-cost camera used for gameplay to perform motion capture. By eliminating the need for depth cameras, inertial sensors, or external hardware, MediaPipe provides an accessible, scalable, and cost-effective foundation for automated rehabilitation assessment.

**Fig. 6:**
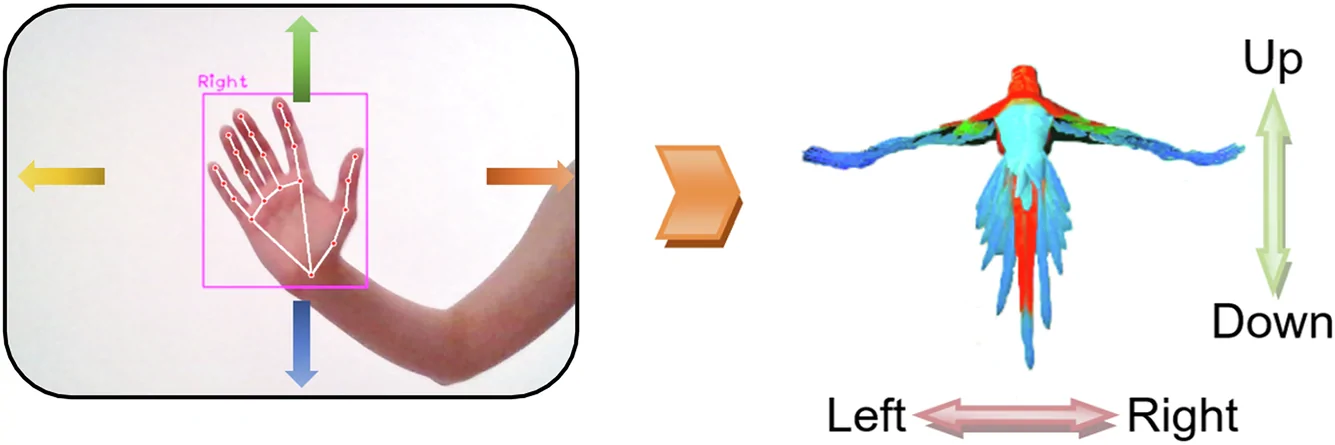
Representation of the hand tracking mechanism used in the exergame system. The MediaPipe framework detects 21 hand landmarks, from which the wrist X and Y position (landmark 0), in pixel units, is extracted to control the avatar’s movement, enabling navigation within the virtual environment. Source: The authors. Edited using Microsoft Word.

Specifically, the X and Y coordinates (in pixels) of the wrist landmark are mapped to the character’s horizontal and vertical movement axes within the virtual environment (Fig. 6).

The bird’s velocity increases proportionally to the wrist’s distance from the screen center, thereby stimulating participants to explore a larger range of motion.

This two-dimensional control strategy was selected to resemble functional components commonly observed in clinical motor evaluations, particularly those involving planar reaching and positioning tasks, such as lifting the arm toward a target or bringing the hand to the mouth. This design not only facilitates the interpretation of gameplay-derived metrics in relation to clinical scores, but also ensures greater accessibility for individuals with reduced motor capacity or fatigue.

A key advantage of this tracking method is the elimination of wearable sensors or physical markers, which simplifies the setup and can reduce implementation costs.

Participants were instructed to maintain the hand extended and parallel to the camera, which allowed the system to extract features such as mean finger extension and range of motion, while ensuring hand visibility for tracking.

### Clinical scale

**Fig. 7:**
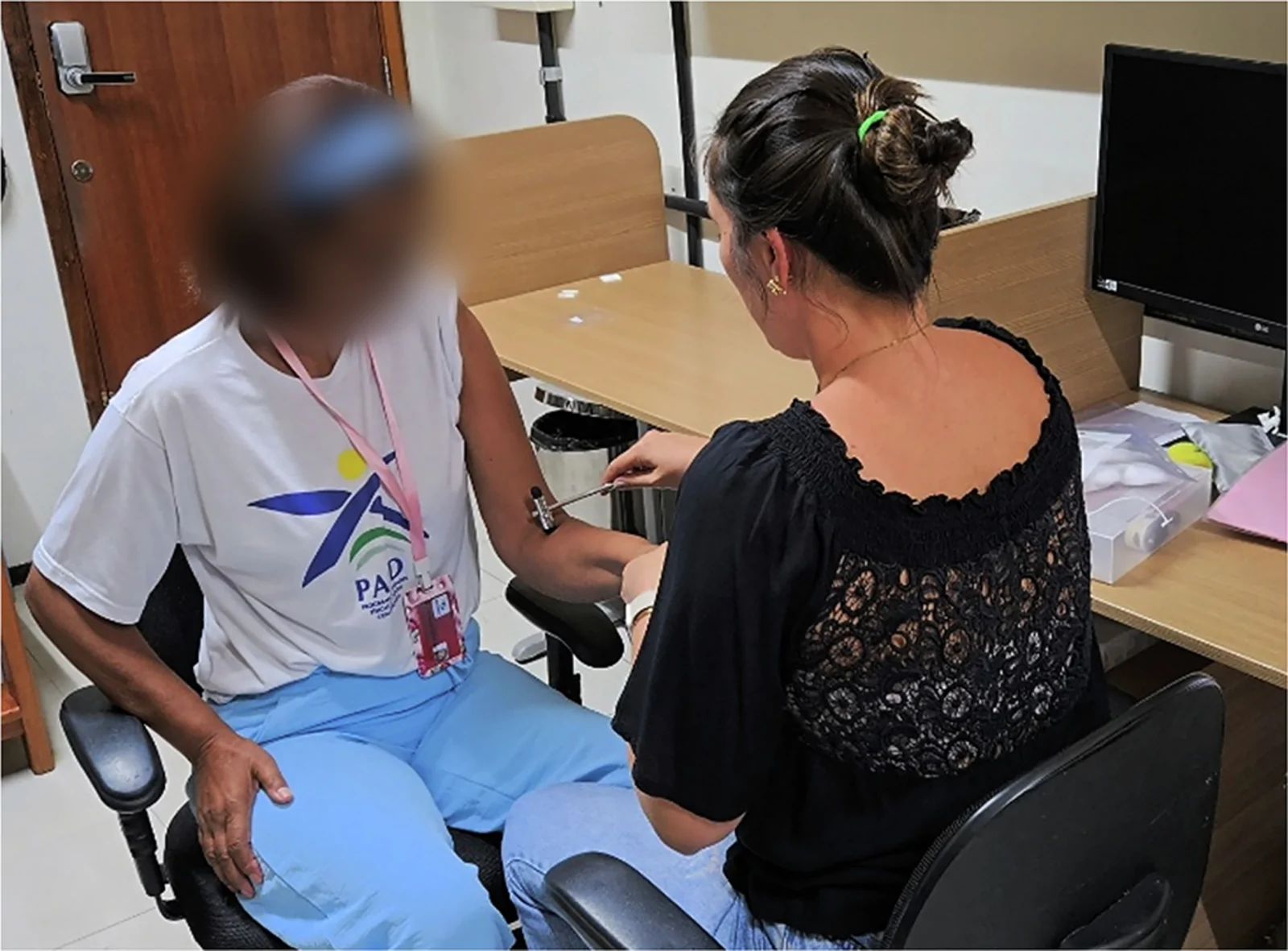
Administration of the FMA. A trained physical therapist evaluates motor function in a post-stroke participant through standardized tasks involving the shoulder, elbow, forearm, wrist, and hand. The assessment was conducted bilaterally in a controlled clinical setting. Source: The authors. Written consent to publish the image was obtained from the participant.

The FMA^24^ is a well-established clinical instrument commonly employed to assess motor deficits in individuals recovering from stroke. It comprises a set of items that examine reflexes as well as isolated and synergistic movements across various segments of the upper limb, including the shoulder, elbow, forearm, wrist, and hand. The total score ranges from 0 to 66, with higher values indicating superior motor performance. In this study, the FMA was applied bilaterally, on both the impaired and non-impaired upper limbs, to enable intra-individual comparisons and to evaluate asymmetries in motor function (Fig. 7). All FMA assessments were conducted by a single licensed physical therapist with more than ten years of experience in neurorehabilitation. The evaluator followed standardized scoring procedures and was blinded to the gameplay and data processing results to minimize bias. Written informed consent for publication of the images was obtained from all participants.

### Data collection

All evaluations were performed during a single experimental session to ensure consistent testing conditions, minimize fatigue or learning effects, and avoid external variables that could compromise data reliability. To ensure that participants were assessed in their baseline motor conditions, the FMA was administered before the exergame task.

To minimize potential bias in the statistical analyses, the application of the FMA was blinded to the individual responsible for data processing and feature extraction.

Each eligible participant completed three minutes of two consecutive gameplay sessions, one using the affected upper limb and another using the unaffected limb.

During gameplay, the system captured data continuously and frame by frame, as follows:

- The 3D coordinates (X, Y, Z) of the 21 hand landmarks detected by the MediaPipe Hands framework;
- The shoulder and elbow joint angles, estimated from the MediaPipe Pose landmarks;
- The game score, defined by the number of fruits successfully collected by the avatar;
- The elapsed time since the beginning of the session, in seconds.

All raw gameplay data were exported in .csv format and analyzed using custom Python scripts to extract key kinematic metrics, such as range of motion, movement smoothness, and average velocity.

### Participants

Participants were recruited from the clinical referral network of the Assistive Technology Laboratory at the Federal University of Uberlândia, Brazil, forming a convenience sample. The sample size was selected based on general recommendations for pilot studies^25^, and the results are intended to inform the feasibility of a future validation study rather than support definitive statistical conclusions.

Eligibility criteria included a clinical diagnosis of chronic stroke with upper-limb motor impairment, sufficient cognitive function to understand task instructions, and the ability to provide written informed consent. Initially, 16 individuals were screened for participation. Four were excluded due to severe flaccid paresis (*n* = 2) or cognitive impairment (*n* = 2), resulting in a final sample of 12 post-stroke participants. Since data were collected for both limbs (*n* = 24) and some participants had double hemiparesis, the resulting sample of limbs was *n* = 14 affected and *n* = 10 controls.

All participants provided informed consent before taking part in the study. The research protocol was approved by the institutional ethics committee at the Federal University of Uberlândia, approval number: 39232820.2.0000.5152. All participants provided written informed consent before participation, in accordance with the Declaration of Helsinki. Throughout the assessments, participants were supervised by a licensed physical therapist to ensure safety and compliance.

### Feature extraction

To investigate the relationship between motor performance during gameplay and clinical upper-limb function, a set of kinematic features was extracted from each participant’s gameplay data. They were processed using custom Python scripts. Although MediaPipe provides 3D coordinates, only the X and Y components were used, as the Z-axis does not provide true depth.

Feature definitions were selected according to the therapeutic focus of the exergame and the quality of camera tracking. Shoulder motion played a minor role in the task, and finger aperture velocity was not extracted because the game did not explicitly require rapid finger movement. The elbow, in contrast, represented the main therapeutic joint and a strong clinical indicator of recovery; thus, both angle and angular velocity were analyzed for this segment. Movements poorly captured from the camera viewpoint were excluded to improve signal reliability. Percentile intervals (e.g., 5th–95th or 25th–75th) were empirically tuned to achieve the best correlation with FMA scores, in line with the regression model’s interpretability focus.

The following features were extracted:

**Wrist Jitter (Wrist_Jitter)**. The Wrist Jitter quantifies the instability of movement by measuring frame-to-frame variability in wrist velocity. It is calculated as the standard deviation of the wrist’s 2D velocity across the entire recording session.

**Maximum Wrist Velocity (Max_Wrist_Vel)**. Defined as the mean of the top 5% highest instantaneous 2D velocity values of the wrist, calculated between frames.

**Average Wrist Velocity (Avg_Wrist_Vel)**. This feature quantifies the overall magnitude of wrist movement during the session and is defined as the mean of the instantaneous 2D velocities across all frames.

**2D Area of Movement (2D_Area)**. To quantify the spatial extent of wrist movement during each trial, the range of motion was computed from the wrist’s (X, Y) coordinates.

To reduce the influence of outliers and tracking noise, the range of motion (ROM) was defined using the 5th and 95th percentiles of the wrist position distribution for each axis, as shown in (1).

1 $${{\mathrm{ROM}}}_{{\mathrm{x}}}={P}_{95}\left(x\right)-{P}_{5}\left(x\right),{{\mathrm{ROM}}}_{{\mathrm{y}}}={P}_{95}\left(y\right)-{P}_{5}\left(y\right)$$

The 2D Area of Movement was then calculated as the product of the horizontal and vertical ROM components, as shown in (2).

2 $${{\rm{Area}}}_{2{\rm{D\; movement}}}=\mathrm{RO}{\mathrm{M}}_{\mathrm{x}}\times \mathrm{RO}{\mathrm{M}}_{\mathrm{y}}$$

Geometrically, this represents the area of the bounding box that encloses the wrist trajectory. This feature reflects the typical amplitude explored by the participant during the task and serves as an indicator of upper-limb mobility and flexibility.

**Total Traveled Distance (Total_Trav_Dist)**. This feature represents the cumulative path length traveled by the wrist throughout the gameplay session, computed in the two-dimensional plane (X, Y). It was obtained by summing the Euclidean distances between consecutive wrist positions, as shown in (3).

3 $${D}_{{\mathrm{total}}}=\mathop{\sum }\limits_{i=1}^{n-1}\left|\vec{{p}_{i+1}}-\vec{{p}_{i}}\right|,{\mathrm{where}}\vec{{p}_{i}}=\left({x}_{i},{y}_{i}\right)$$

Here, $\vec{{p}_{i}}$ is the 2D position of the wrist at frame i, and n is the number of frames. This metric reflects both movement amplitude and frequency, capturing the total excursion of the wrist during the task.

**Average Time-to-Target (Avg_Time_Target)**. For every successful capture, the time-to-target is defined as the interval between the start of the movement segment and the moment the fruit is collected.

**Total Score (Score)**. The total score is the total number of fruits collected at the end of the session. This feature represents overall task performance, as more collected fruits can indicate better performance and coordination.

**Average Hand Angle (Avg_Hand_Angle)**. This feature quantifies the average angle of hand opening and closing throughout the session by computing the flexion angles of each finger. For each frame, the flexion angle of a finger is defined as the angle formed by three anatomical landmarks: the metacarpophalangeal (MCP) joint, the proximal interphalangeal (PIP) joint, and the fingertip (TIP). The angle is calculated as shown in (4),

4 $${{\rm{\theta }}}_{\mathrm{finger}}={\cos }^{-1}\left(\frac{\vec{a}\cdot \vec{b}}{|\vec{a}{||}\vec{b}|}\right)$$

where $$\vec{a}=\mathrm{PIP}-\mathrm{MCP}$$ and $$\vec{b}=\mathrm{TIP}-\mathrm{PIP}$$.

For each frame, the average flexion angle across the five fingers is computed, as shown in (5).

5 $${{\rm{\theta }}}_{{\mathrm{mean}},i}=\frac{1}{5}\mathop{\sum }\limits_{j=1}^{5}{{\rm{\theta }}}_{j,i}$$

Then, a single global metric is derived by averaging ${\theta }_{\mathrm{mean},i}$ across all valid frames. This final value reflects the typical level of finger flexion exhibited during the task and may indicate voluntary control over hand opening and closing.

**Average Shoulder Angle (Avg_Shoulder_Angle)**. The shoulder angle is defined as the angle formed between the trunk (hip-to-shoulder vector) and the upper arm (shoulder-to-elbow vector) at each frame. The average is then computed across all valid frames.

**Average Elbow Angle (Avg_Elbow_Angle)**. This feature is computed frame-by-frame using the angle formed between the upper arm (shoulder-to-elbow vector) and the forearm (elbow-to-wrist vector), extracted from MediaPipe landmarks.

**Average Elbow Angular Velocity (Avg_Elbow_Vel)**. This feature is defined as the mean of the instantaneous angular velocities across all frames.

**Maximum Elbow Angular Velocity (Max_Elbow_Vel)**. This feature is computed as the mean of the top 5% highest instantaneous angular velocities of the elbow observed throughout the session.

This metric serves as a indicator of the participant’s ability to generate rapid, forceful elbow motions, often linked to motor control efficiency and residual strength.

**Shoulder Angle Range of Motion (ROM_Shoulder_Angle)**. This feature quantifies the typical angular excursion of the shoulder during the session by computing the interquartile range of the shoulder angle distribution. Specifically, it is defined as the difference between the 75th and 25th percentiles.

**Elbow Angle Range of Motion (ROM_Elbow_Angle)**. This feature is calculated as the interquartile range of the elbow angle distribution, capturing the central 50% of values and excluding extremes.

**Hand Angle Range of Motion (ROM_Hand_Angle)**. This metric estimates the total angular excursion of the hand by capturing the range of average finger flexion angles throughout the session. It is computed as the difference between the 95th and 5th percentiles of the hand flexion angle distribution, as shown in (6):

6 $${\mathrm{ROM}}_{\mathrm{hand}}={P}_{95}\left({\theta }_{\mathrm{hand}}\right)-{P}_{5}\left({\theta }_{\mathrm{hand}}\right)$$

where ${\theta }_{\mathrm{hand}}$ denotes the average flexion angle across all fingers for each frame.

Larger values reflect greater capacity for opening and closing the hand, which is relevant to tasks involving grasp and release. Reduced values may indicate spasticity, joint restriction, or lack of voluntary finger extension.

**Shoulder-Elbow Correlation (Corr_Shoulder_Elbow)**. This feature is computed using the Pearson correlation coefficient between the shoulder and elbow angles across all valid frames, as shown in (7):

7 $${\mathrm{Coord}}_{\mathrm{shoulder}-\mathrm{elbow}}=\mathrm{corr}\left({\theta }_{\mathrm{shoulder}},{\theta }_{\mathrm{elbow}}\right)$$

This value ranges from −1 (perfectly inverse coordination) to +1 (perfectly direct coordination), with values near zero indicating low or inconsistent coupling between the two joints.

Higher correlations may reflect synergistic or stereotyped movement patterns, commonly observed in early or moderate post-stroke recovery phases. Lower values may indicate decoupled joint control, which can emerge with greater motor recovery or impaired coordination. This feature is particularly informative in distinguishing between patients who rely heavily on synergy patterns versus those with more isolated joint control.

### Statistical analysis

All analyses were performed using Python 3.11 (SciPy, scikit-learn, and Pandas). To provide an overview of the distribution of each digital feature across severity groups, standardized values (Z-scores) were visualized using a boxplot. This visualization allows for the inspection of descriptive statistics, including the median, standard deviation, interquartile range, and potential outliers.

In addition to statistical feature comparisons, the full sequence of wrist’s (X, Y) coordinates was extracted from the gameplay recordings, and overlaid plots were generated by severity group. These plots aimed to qualitatively assess movement range, directionality, and dispersion across groups.

The normality of all variables was assessed using the Shapiro-Wilk test. If the majority of variables demonstrated normal distribution, Pearson’s correlation coefficient was used to evaluate the linear association between each feature and the FMA score. Otherwise, Spearman’s rank correlation coefficient was used. The significant features were ranked by the correlation coefficient found and presented in a correlation heatmap.

To assess the predictive value of gameplay-derived features, multiple linear regression models were built using FMA scores as the dependent variable and up to five features selected via exhaustive feature selection. All features were standardized using Z-score normalization.

Each limb (affected and non-affected) was treated as an independent observation because they represent distinct functional states with distinct FMA scores and were recorded in separate sessions. Only two participants presented bilateral impairment, and each limb exhibited different degrees of motor deficit. This approach expands the range of FMA scores while maintaining independence between samples. Although not explored here, this design also enables future intra-personal analyses that may control for cognitive or behavioral variability by comparing both limbs of the same individual. Also, this strategy helps reduce variability related to inter-individual cognitive and behavioral differences.

Models were trained and evaluated separately for the affected and total limb groups. Two validation strategies were used: hold-out (50/50 for affected, 70/30 for total) to simulate real-world generalization with approximately 7 participants in the test set, and leave-one-out cross-validation (LOOCV) to maximize data usage and minimize overfitting, especially in small samples.

Model performance was based on the highest Spearman correlation (ρ), lowest root mean square error (RMSE), or highest coefficient of determination (R²) between predicted and observed FMA values. A scatter plot illustrates the agreement between predicted and true FMA scores for the best model in the affected group.

Given the pilot nature of the dataset and the small number of participants per severity subgroup (particularly mild, *n* = 3), regression models were trained on the total (*n* = 24) and affected-limb (*n* = 14) datasets to ensure broader representation and mitigate class imbalance.

### Diagnostic accuracy evaluation

To assess whether predicted FMA values could be used to support clinical classification of motor severity, an exploratory diagnostic accuracy analysis was conducted. The best regression equation identified in the previous analysis was applied to the affected limb subgroup (*n* = 14) data.

The input features were Z-score normalized to ensure consistency with the coefficients used. Real and predicted FMA scores were then categorized into three clinical severity levels based on established thresholds: (i) severe impairment (FMA ≤ 20), (ii) moderate impairment (21 ≤ FMA ≤ 45), (iii) mild impairment (FMA > 45).

**Fig. 8:**
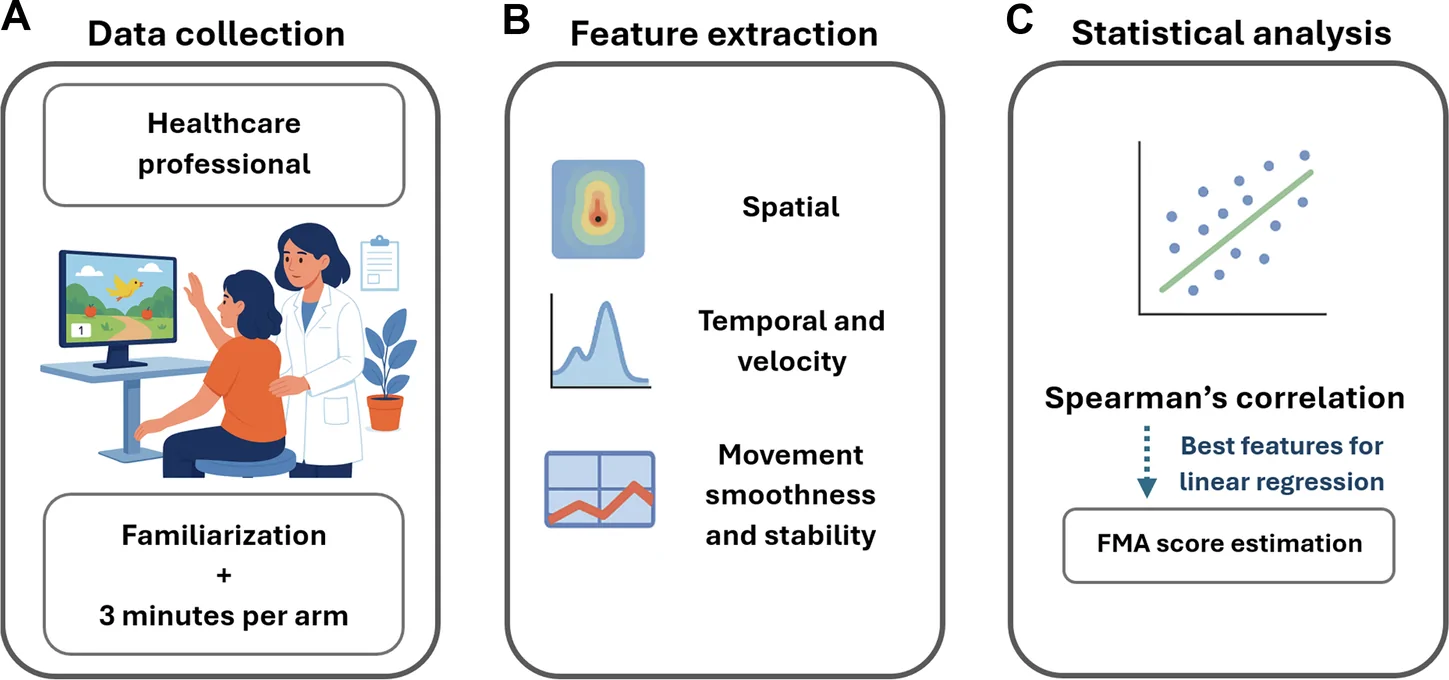
Overview of the data processing pipeline. The workflow encompasses three main phases: **A** (left panel) data collection during gameplay with clinical supervision; **B** (central panel) feature extraction of spatiotemporal and kinematic metrics; and **C** (right panel) statistical modeling to examine correlations with upper-limb motor function as measured by the FMA. Source: The authors. Image elements generated with ChatGPT-5; final composition and text edited in Microsoft Word.

A confusion matrix was computed comparing predicted classes to ground truth labels. Subsequently, class-wise precision, recall, F1-score, and accuracy were calculated. Figure 8 shows an overview of the data processing pipeline.

### Machine learning analysis

To explore different predictive strategies, four machine learning models were evaluated using a 70/30 hold-out split and Z-score normalization in the total dataset. Performance was assessed with R², RMSE, and Spearman’s ρ.

The Random Forest was trained on six pre-extracted kinematic features strongly associated with FMA. Using pre-extracted features was justified because Random Forest excels in handling small sample sizes and offers interpretable variable importance, making it well-suited for datasets with limited observations.

In contrast, the 1D-CNN + Bidirectional LSTM model was trained directly on raw time-series hand coordinates (100 Hz). This choice leveraged the ability of deep learning to automatically extract complex temporal patterns from large amounts of sequential data, avoiding the need for manual feature engineering. Predictions from multiple windows were averaged to yield a single FMA estimate per arm.

Finally, Lasso and Elastic Net regressions were applied to the same raw windowed data. These models served as simpler, more interpretable baselines while still allowing direct comparison with the deep learning approach.

Machine learning models were implemented as exploratory baselines to assess feasibility and scalability rather than to provide fully optimized benchmarks. Random Forest was trained on pre-engineered kinematic features due to their stability and transparency in low-sample scenarios, while the other networks were trained on raw time-series data to test the feasibility of end-to-end learning. All models used identical data splits and normalization pipelines to ensure fair comparison. No definitive conclusions regarding model superiority can be drawn due to sample size limitations.

## Data Availability

The datasets generated and analyzed during the current study are not publicly available at this stage but are available from the corresponding author (J.T.) upon reasonable request. We also intend to make the anonymized dataset publicly accessible through an open research repository following publication. The custom Python scripts used for data preprocessing, feature extraction, and regression analysis are available from the corresponding author upon request.
